# Antioxidative and Cytoprotective Effects of *Rosa Roxburghii* and Metabolite Changes in Oxidative Stress-Induced HepG2 Cells Following *Rosa Roxburghii* Intervention

**DOI:** 10.3390/foods13213520

**Published:** 2024-11-04

**Authors:** Yangchen Mao, Ruyi Sha, Yuhao Sun, Zhenzhen Wang, Jun Huang

**Affiliations:** 1 Zhejiang Provincial Key Laboratory for Chemical & Biological Processing Technology of Farm Product, Hangzhou 310023, China; m13735877964@163.com (Y.M.);; 2 Zhejiang Province Collaborative Innovation Center of Agricultural Biological Resources Biochemical Manufacturing, School of Biological and Chemical Engineering, Zhejiang University of Science and Technology, Hangzhou 310023, China

**Keywords:** *Rosa Roxburghii*, HepG2, oxidative stress, antioxidants, natural products

## Abstract

*Rosa Roxburghii* (RR), a traditional Chinese medicinal fruit, is rich in bioactive substances that make it a potential natural antioxidant resource. This research aimed to study the antioxidant properties of RR by in vitro experiments and through intracellular assessment in H_2_O_2_-induced HepG2 cells. A non-targeted metabolic analysis was conducted to indicate changes in intracellular and extracellular metabolites. Differential metabolites and metabolic pathways were explored using PCA, PLS-DA, and KEGG pathway analysis. The results showed that RR rich in bioactive substances exhibited a significant antioxidative property in vitro and intracellularly. This property may be achieved by scavenging free radicals, increasing the activity of catalase (CAT), glutathione peroxidase (GSH-Px), superoxide dismutase (SOD), and the levels of bicinchoninic acid (BCA) while reducing the reactive oxygen species (ROS) generation. This study identified 13 differential metabolites intracellularly and 7 extracellularly, among which the key differential metabolites included D-glucopyranose, D-mannose, fructose, citric acid, malic acid, cholesterol, and cholestenone. These key metabolites primarily regulated glucose-related metabolism, the citrate cycle, and the primary bile acid biosynthesis pathway in H_2_O_2_-induced HepG2 cells. These findings provide potential application evidence of RR in the development of natural resources for functional foods.

## 1. Introduction

Aerobic organisms possess a unique antioxidant defense system, but excessive generation of reactive oxygen species (ROS) can exceed the defense capacity and lead to oxidative stress [1,2]. Moderate ROS promote cell signaling, but excessive ROS cause oxidative damage, affecting the cell cycle and differentiation, cancer proliferation, and biomolecule damage [2]. ROS lead to health problems such as degenerative diseases, cardiovascular disease, diabetes, liver damage, cancer, and obesity by disrupting cellular function [2,3,4,5,6,7,8]. The mechanisms of ROS generation are complex, including intracellular organelles, pro-oxidant enzymes, detoxification, and lipid peroxidation [9,10]. Its complexity is influenced by various endogenous (immune response, inflammation, etc.) and exogenous (pollution, drug side effects, etc.) factors [11]. Improving endogenous antioxidant defenses, especially by upregulating antioxidant enzymes and non-enzymatic antioxidants, is a strategy to reduce ROS-induced damage [12]. This can be achieved through dietary intake of antioxidants, for example, phenols, flavonoids, vitamin C, and SOD, which stimulate the up-regulation of endogenous antioxidant enzyme activity [11,12,13,14].

Although natural antioxidants exhibit promising applications, the employment of synthetic antioxidants in food processing continues to be prevalent [15]. However, the long-term use of synthetic antioxidants may pose potential risks to biological health, which still cannot be overlooked [16]. For example, butylated hydroxyanisole (BHA) may be neurotoxic, interfere with sex hormone levels, and have potential estrogenic effects, whereas butylated hydroxytoluene (BHT) has cardiotoxicity, and propyl gallate (PG) may induce oxidative damage to DNA [16,17,18,19,20,21]. Based on the potential health risks associated with synthetic alternatives, researchers are increasingly exploring natural plant actives, which show greater application due to their antioxidant function and biocompatibility.

*Rosa Roxburghii* (RR), a member of the *Rosaceae* family, has mature fruit covered with dense yellow awns and a thick pericarp [22]. RR grows in the subtropical regions of the southwestern and central provinces of China, especially in the dense scrub forests of Guizhou, Yunnan, and Sichuan, on the fringes of mountains or plateaus and has become a specialty plant of these regions [23]. RR is a plant traditionally used for food and medicine in China, with a long tradition of consumption since the end of the 17th century, and it has been used in historical medicine for intestinal diseases [24]. Several in vitro and animal research studies have evidenced that RR can prevent type II diabetes, ovarian cancer, etc. [25,26]. It also has functional activities such as reducing inflammation, α-glucosidase inhibitory activity, anti-apoptosis, antimicrobial properties, inhibiting thrombosis, and protecting hepatic lipid metabolism stability [24,27,28,29,30]. The wide range of functions of RR relies on its richness in nutritional and functional components, such as vitamins (C, B, E, P), polyphenol compounds, amino acids, organic acids, and minerals [31]. In particular, RR has a strong antioxidant capacity due to the effects of phenolic compounds, polysaccharides, L-ascorbic acid, and SOD [22,23,24,32]. Specifically, the content of L-ascorbic acid in fresh RR fruits is as high as 54,000 U/100 mL, which is 10 to 20 times higher than that in kiwifruit [24,33]. The broad range of active functions of RR has attracted research attention, with previous studies focusing on the functional activity substances of RR constituents and a limited number of studies having delved into the metabolomics of the effects of RR intervention on cellular metabolomics effects.

H_2_O_2_, a commonly used oxidative stress inducer, has been used to mimic oxidative stress conditions in HepG2 cells, allowing for the study of both liver functions and oxidative stress. Furthermore, it was previously found that genotype affects the metabolic profile of RR, which may have an impact on its antioxidant properties [34]. Therefore, this study quantified the concentration of active substances present in the RR used and verified its antioxidant properties using in vitro and cellular experiments. To address the gap in previous studies, this study analyzed the changes in intracellular and extracellular metabolites of H_2_O_2_-injured HepG2 cells after RR interventions. The findings provide strong theoretical support for the potential application of RR, a natural antioxidant plant, in the field of functional food development and provide a rationale for the protection of a traditional plant resource, which is expected to be an ingredient for future health food innovation.

## 2. Materials and Methods

### 2.1. Materials and Chemicals

RR was provided by Wanyuanjia Pharmaceutical (Chongqing, China). Superoxide Dismutase (SOD), bicinchoninic acid (BCA) assay kits, and RIPA lysis buffer were provided by Jiancheng Bioengineering Institute (Nanjing, China). N-Methyl-N-(trimethylsilyl) trifluoroacetamide (MSTFA), Coomassie Brilliant Blue G250 (CBB), hydrochloric acid (HCl), tris-HCl buffer, aluminum nitrate, bovine serum albumin (BSA), dimethyl sulfoxide (DMSO), isopropanol, and single standard acid were bought from Macklin (Shanghai, China). HepG2 cells were obtained from Zhejiang University. Folin–Ciocalteu reagent (FCR) and phenol were bought from Sinopharm (Shanghai, China). Gallic acid, rutin, phosphoric acid, methanol, L-2 chlorophenylalanine, and H_2_O_2_ solution were bought from Aladdin (Shanghai, China). Phosphate buffer solution (PBS), penicillin–streptomycin solution (100×), Dulbecco’s Modified Eagle Medium (DMEM), and serum of fetal bovine (SFB) were bought from Procell Life Science & Technology (Wuhan, China). Catalase (CAT) and Glutathione Peroxidase (GSH-Px) assay kits and 2′,7′-Dichlorodihydrofluorescein diacetate (DCFH-DA) were bought from Beyotime (Shanghai, China). All remaining chemicals were purchased from Lingfeng Chemical Reagent (Shanghai, China).

### 2.2. Preparation of Rosa Roxburghii Aqueous Extract

Fresh RR was harvested in September 2023 in Guizhou Province. It was sun-dried and stored at room temperature. The extraction method was based on that of Mu et al. [35] with slight modifications. The RR was crushed and then dissolved in water (RR/W = 1/9, m%) using an ultrasonic extraction method (400 W, 40 kHz, 1 h) to obtain RR ultrasonic aqueous extract (RRE).

#### 2.2.1. Total Phenol Content

The RRE (50 μL) was dissolved to a volume of 0.5 mL by adding deionized water, and then FCR (2.5 mL, 10%, v%) and sodium carbonate solution (2.0 mL, 7.5%, w%) were added sequentially. The reaction was protected from light (25 °C, 1 h). With deionized water as the reference solution, the total phenol was expressed as gallic acid equivalent (μg GAE/mL), as determined at the absorbance value (at 765 nm wavelength).

#### 2.2.2. Total Flavonoid Concentration

Sodium nitrite solution (0.1 mL, 5%, m%), aluminum nitrate solution (0.1 mL, 10%, m%), NaOH solution (0.2 mL, 4%, m%), and deionized water (1.5 mL) were added to the RRE (0.6 mL), sequentially. With deionized water as the reference solution, the total flavonoid was expressed as rutin equivalents (mg RE/mL), as determined at the absorbance value (at 510 nm wavelength).

#### 2.2.3. Soluble Protein Concentration

BSA (25 mg) was diluted to 250 mL using deionized water to obtain standard protein solutions (SPSs). The standard protein solutions were pipetted into distilled water to achieve fixed concentrations (0.01, 0.02, 0.04, 0.06, 0.08, 0.1 mg/mL). In addition, CBB solution was prepared as follows: CBB (100 mg) was dissolved in 95% ethanol (50 mL), followed by the addition of 85% phosphoric acid (100 mL), and finally diluted and volume-fixed to 1000 mL with distilled water. The CBB solution (5 mL) was mixed with 6 different concentrations of SPS. The absorbance values (at 595 nm) of each mixture were recorded, and standard curves were plotted. Finally, CBB solution (5 mL) was added to the RRE (1 mL). The absorbance value of each solution (at the same wavelength) was measured.

#### 2.2.4. Amino Acid Concentration

First, GABA (50 μL, 10 mmol/mL) was added to 200 μL of amino acid standard solution, and then HCl (0.02 mol/L) was used to adjust the volume to 5 mL in order to obtain a mixed standard solution (100 nmol/mL). The standard solution (18-amino acid mixture) was diluted to the concentrations of 0.01, 0.025, 0.05, 0.075, 0.1, and 0.2 μmol/mL with deionized water. The RRE (1 mL) was pipetted into hydrolysis tubes, and HCl (10 mL, 6 mol/L) and phenol (3 drops) were subsequently added. Then, the mixture was cooled, filled with high-purity nitrogen, and put into a drying oven for hydrolysis (110 °C, 22 h). The hydrolysate was then diluted with distilled water to a volume of 25 mL and filtered through a filter membrane (0.22 μm). The filtered hydrolysate (1 mL) was taken and dried (45 °C) to obtain a solid residue. The residue was dissolved in distilled water (1 mL) and dried again (45 °C). The dried solid was dissolved using HCl (1 mL, 0.02 mol/L) and then filtered through a filter membrane (0.22 μm). The amino acid content was determined at 440 nm and 570 nm wavelengths using a Hitachi L8900 automatic amino acid analyzer (Hitachi High Technologies Corporation, Tokyo, Japan) with an injection volume of 20 μL. The analyzer was set according to the manufacturer’s instructions.

#### 2.2.5. Organic Acid Concentration

The organic acid standards were dissolved in deionized water to ensure that the final concentration of each standard was 1 mg/mL to obtain a single standard solution. Each single standard solution was mixed so that the mass fraction of each organic acid in the mixed solution reached 100 μg/mL of oxalic acid, 25 μg/mL of fumaric acid, 500 μg/mL of succinic acid, 500 μg/mL of lactic acid, 5 μg/mL of gallic acid, 1000 μg/mL of acetic acid, and 25 μg/mL of ascorbic acid. The mixed solution was filtered using a microporous filter membrane (0.22 μm), and the content was measured using High-Performance Liquid Chromatography (Waters e2695, PDA, XBridge C18, Waters, Milford, MA, USA) at a wavelength of 215 nm.

#### 2.2.6. Total Sugar and Polysaccharide Concentration

The preparation of solutions for plotting the standard curve involved taking volumes of 0, 20, 40, 60, 80, and 100 μL of standard glucose solutions (1 mg/mL) and adjusting their volumes to 1 mL each with deionized water. The RRE was diluted 500-fold, and 200 μL was taken for subsequent experiments. Phenol solution (1 mL, 1 mg/mL) and concentrated H_2_SO_4_ (5 mL) were added to the six concentrations of standard glucose solutions and the diluted RRE solution. The absorbance at 490 nm was then measured to draw the standard curve and calculate the total sugar concentration.

To prepare the standard curve for reducing sugar, 0, 100, 200, 300, 400, and 500 μL of the standard glucose solution (1 mg/mL) were taken and diluted with water to achieve a total volume of 500 μL for each. The RRE was diluted 100-fold, and 500 μL of the diluted solution was taken for subsequent experiments. To these diluted solutions, DNS solution (1 mL) was added, followed by heating in a water bath (10 min). After cooling, deionized water (4.5 mL) was added to each solution. Subsequently, the absorbance of each solution was recorded at 540 nm to plot the standard curve and calculate the reducing sugar content. The polysaccharide concentration was measured by determining the difference between the soluble sugar concentration and the obtained reducing sugar content.

#### 2.2.7. SOD Activity

SOD activity was measured using the SOD kit by referring to the product instructions.

### 2.3. In Vitro Antioxidant Ability

#### 2.3.1. ABTS+ Free Radical Scavenging Ability

Different times of the RRE were diluted in PBS (5 mmol/L, pH 7.4). The diluted solution (200 μL) was added to the ABTS radical PBS solution (1 mL, 7 mmol/L) and allowed to react (30 °C, 1 h). Absorbance (at 734 nm) was measured with deionized water as the reference.

#### 2.3.2. DPPH Radical Scavenging Ability

Different times of the RRE were diluted in deionized water. The solutions (0.5 mL) were added to DPPH–methanol solution (1 mL, 0.1 mmol/L) and tris-HCl buffer (100 μL, 50 mmol/L, pH 7.4) sequentially and reacted in a water bath (25 °C, 30 min). Absorbance (at 517 nm) was determined using deionized water as the reference.

#### 2.3.3. Hydroxyl Radical Scavenging Ability

Different concentrations of the RRE were diluted in deionized water. The solutions (200 μL) were added to H_2_O_2_ solution (140 μL, 6 mmol/L), sodium salicylate solution (60 μL, 20 mmol/L), and ferrous sulfate solution (200 μL, 1.5 mmol/L) and then put in a water bath (37 °C, 1 h). Absorbance (at 510 nm) was determined using deionized water as the reference.

### 2.4. Cell Culture

HepG2 cells were cultured in a specific environment (CO_2_ concentration of 5%) using DMEM with penicillin–streptomycin (10% content) and SFB (1% content) for 3 passages. Using 96-well plates (100 μL, 1 × 105 cells/mL), HepG2 cells were inoculated (37 °C, 24 h).

### 2.5. The Cell Viability of the RRE Intervention and H_2_O_2_-Induced HepG2 Cells

The RRE was diluted twofold from an initial concentration of 200 μL/mL. The diluted RREs were treated to the cells described in Section 2.4 (under treatment conditions of 24 h at 37 °C), resulting in three concentrations of RRE (TCR) that did not damage the cells. Based on Section 2.4, different concentrations of H_2_O_2_ medium solution culture (2 h) were added to establish the oxidative stress injury HepG2 model. Using the MTT method [36], cell viability was assessed by measuring the absorbance (at 570 nm).

### 2.6. Experimental Grouping and Treatments

Based on Section 2.4, the medium was aspirated to set up the control group, the model group, and the experimental groups (low-, moderate-, and high-concentration). The control and model groups were treated with DMEM (24 h), and the experimental groups were treated with TCR (24 h). The DMEM or samples were then aspirated from all groups. Then, the control groups were treated with DMEM (2 h), and the model group and the 3 experimental groups were treated with semi-inhibitory concentrations of H_2_O_2_ solution (2 h). Finally, DMSO solution (100 μL) was added to each well after the medium was aspirated. The cell viability was measured in the same way as in Section 2.5.

### 2.7. Biochemical Indicators of H_2_O_2_-Induced HepG2 Cells

#### 2.7.1. Cellular Antioxidant Enzyme Activity and BCA Level Measurements

Cells from the five groups were inoculated into 6-well plates (2.5 mL, 5 × 105 cells/mL). The cells were rinsed with PBS; the process was repeated 3 times, and then RIPA lysis buffer (250 μL) was added to each well. Finally, SOD, CAT, and GSH-Px activities and BCA levels were measured with the kit.

#### 2.7.2. Intracellular ROS Level Measurement

Cells from the five groups were inoculated into 24-well plates (1 mL, 5 × 105 cells/mL). Afterward, DCFH-DA solution (1 mL, 10 µmol/L) was added to each well (treated for 30 min), and then the solution was aspirated. DMEM without SFB was used to wash the cells 3 times, and then PBS (1 mL) was added to the wells. Finally, the absorbance (excitation wavelength, 500 nm; emission wavelength, 525 nm) was measured.

### 2.8. Cellular Metabolite Extraction

To obtain extracellular metabolites, cells from the five groups were inoculated in a 6-well plate (5 × 106 cells/mL, 2 mL). Methanol (150 μL) and L-2 chlorophenylalanine (20 μL, 8 mg/mL) were added to the cell supernatant medium (150 μL) of each of the 5 groups and then finally stored (−80 °C).

To obtain intracellular metabolites, the 5 groups of cells were washed 3 times with PBS. A methanol–isopropanol solution (1 mL, 3/1, v%) was added to the precipitated cells, and the resulting mixture was sonicated at 4 °C for 20 min (40 kHz, 500 W). The solution was frozen in liquid nitrogen and then thawed at room temperature; this process was repeated 5 times. The solution was processed by centrifugation (13,000 rpm, 15 min, 4 °C), and the supernatant (300 μL) was pipetted into EP tubes, to which L-2 chlorophenylalanine (20 μL, 8 mg/mL) was added and then finally stored (80 °C).

### 2.9. Derivatization

Intracellular and extracellular metabolite extracts were concentrated and dried. Methoxypyridine solution (80 μL, 20 mg/mL) was added, thoroughly mixed, and incubated (80 °C, 20 min). This was followed by the addition of MSTFA (100 μL), mixing and incubation (70 °C, 60 min), and centrifugation (13,000 rpm, 4 °C, 15 min).

### 2.10. GC-MS Condition

The GC-MS instrument (Agilent, Santa Clara, CA, USA) was set as a reference based on the study by He et al. [37] with the injection volume (1 μL) and temperature (280 °C). The temperature settings included the transfer line (280 °C), ion source (250 °C), and helium carrier gas (1.0 mL/min). The heating temperature was initiated (40 °C, held for 1 min), followed by the process temperature (ramped up to 310 °C, 8 °C/min), with a final hold temperature (310 °C for 10 min). The results were identified using the NIST 14 standard mass spectrometry database.

### 2.11. Data and Statistical Analysis

After normalizing the data using unit variance scaling, data patterns and differential metabolites were identified through SIMCA 14.1 (Umetrics, Umea, Sweden). Differential metabolites were identified by VIP values >1 and a significant difference between groups of *p* < 0.05. KEGG enrichment analysis of these differential metabolites was carried out using MetaboAnalyst 6.0 (McGill University, Montreal, Canada) [38]. Using SPSS 26 (IBM, Armonk, NY, USA), significant differences between groups were identified by employing the test of Duncan’s Multiple Range, following One-way ANOVA. All data were presented as mean ± SD, while significant differences were determined at *p* < 0.05.

## 3. Results

### 3.1. Active Components

#### 3.1.1. Total Phenols, Flavonoids, Protein, and Superoxide Dismutase (SOD)

This study quantified the active substances in RRE. As shown in Table 1, the total flavonoid concentration of the RRE was 5.10 ± 0.04 mg RE/mL, whereas the total phenol concentration was 0.80 ± 0.13 mg GAE/mL. The results showed that total sugar, polysaccharide concentration, and SOD activity in the RRE amounted to 15.29 ± 0.25 mg/mL, 4.75 ± 0.86 mg/mL, and 81 ± 0.54 U/mL, respectively (Table 1).

#### 3.1.2. Protein, Amino Acids, and Organic Acids

**Figure 1:**
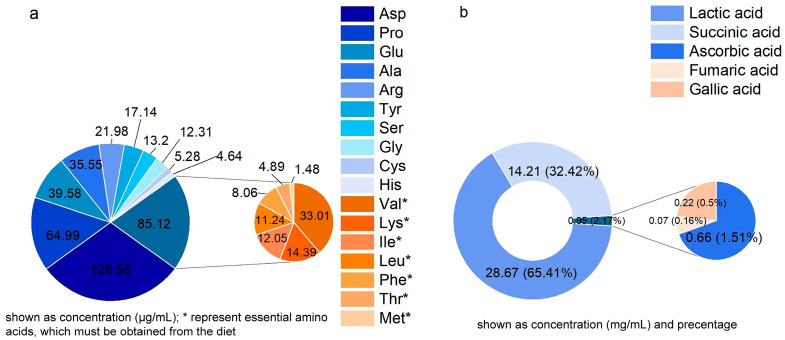
Amino acid (**a**) and organic acid (**b**) concentrations. Asp, Aspartic acid; Pro, proline; Glu, Glutamic acid; Phe, Phenylalanine; Ala, alanine; Arg, arginine; Tyr, Tyrosine; Lys, lysine; Ser, Serine; Gly, Glycine; Cys, Cysteine; Ile, Isoleucine; His, Histidine; Val, valine; Leu, Leucine; Met, Methionine; Thr, Threonine. * represents essential amino acids, which must be obtained from the diet.

This study detected the concentration of soluble protein, amino acids, and organic acids in the RRE. As shown in Table 1 and Figure 1a, the soluble protein concentration was 115.80 ± 5.25 μg/mL, and the total amino acid concentration was 428.33 ± 3.26 μg/mL. Furthermore, the RRE was detected to contain a total of 17 amino acids, among which Asp (128.55 ± 2.25 μg/mL), Pro (64.99 ± 1.35 μg/mL), and Glu (39.58 ± 0.75 μg/mL) were the most abundant. Moreover, the RRE contained seven essential amino acids, including Val, Lys, Ile, Leu, Phe, Thr, and Met, which accounted for 19.87% of the total amino acid content. As shown in Figure 1b, among the five organic acids detected in the RRE, lactic acid had the highest content.

### 3.2. Cellular Cytotoxicity

#### 3.2.1. Cell Modeling

**Figure 2:**
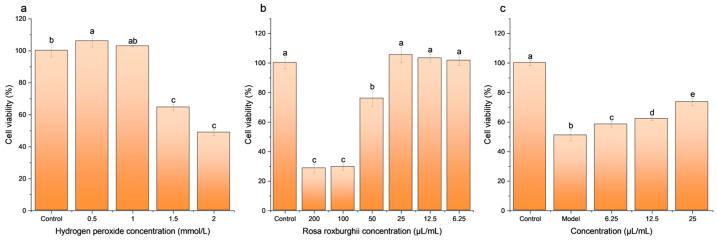
Cell modeling and cell cytotoxicity. (**a**) Cell viability after intervention with different concentrations of hydrogen peroxide. The horizontal coordinates represent four concentrations of hydrogen peroxide, with 2 mmol/L being the semi-inhibitory concentration (SIC). (**b**) Cell viability after intervention with different concentrations of RR. The horizontal coordinates indicate six concentrations of the RRE. (**c**) Cell viability in the control group, model group (SIC), and experimental groups. Different letters indicate a significant difference between groups, *p* < 0.05.

Figure 2 shows the cell viability of different concentrations of H_2_O_2_-induced HepG2 cells. The viability of HepG2 cells significantly increased at a concentration of 0.5 mmol/L H_2_O_2_ (105.96 ± 3.59%) and gradually decreased with increasing H_2_O_2_ concentration (from 105.96 ± 3.59% to 48.76 ± 1.73%). Specifically, at a H_2_O_2_ concentration of 2 mmol/L, the cell viability was 48.76%, which was the closest to half the maximal inhibitory concentration of the viability. Therefore, the concentration of 2 mmol/L H_2_O_2_ was employed to establish the oxidative damage model in HepG2 cells.

#### 3.2.2. Cytotoxicity

As shown in Figure 2b, the cellular cytotoxicity of different concentrations of the RRE was assessed. Three concentrations of the RRE showed no significant difference in cell viability compared to the control group, suggesting that the RRE was not cytotoxic to cells at concentrations of 25 μL/mL, 12.5 μL/mL, and 6.25 μL/mL. The cell viability was 105.41% ± 5.29%, 103.27% ± 2.75%, and 101.54% ± 3.28%, respectively. Therefore, 25 μL/mL (high-concentration group, H), 12.5 μL/mL (moderate-concentration group, Mo), and 6.25 μL/mL (low-concentration group, L) were used for the following experiments.

The results of the H_2_O_2-_induced HepG2 cell intervention with TCR are shown in Figure 2c. Comparing the experiment groups to the model group, the cell viability (from 50.94 ± 3.71% to 73.51 ± 2.42%) of each RRE-treated group was significantly increased (*p* < 0.05). Furthermore, the cell viability increased with the concentration of the RRE, and the most significant protective effect was observed at a concentration in the high group (73.51 ± 2.42%), followed by the moderate group (62.17 ± 1.20%) and the low group (58.35 ± 1.76%) (*p* < 0.05). The results showed that RRE was effective in restoring HepG2 cell viability from H_2_O_2_-induced oxidative damage within a specific concentration range, suggesting that its restorative ability was concentration-dependent.

### 3.3. Antioxidant Activity

#### 3.3.1. In Vitro Radical Scavenging Capacity

**Figure 3:**
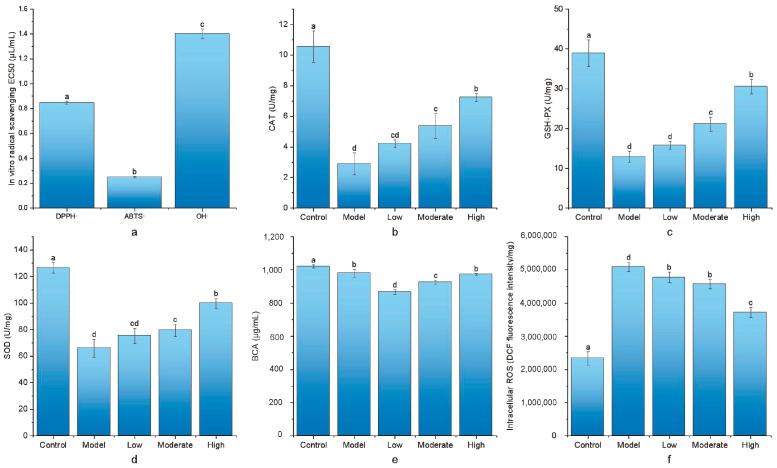
In vitro cellular antioxidant enzyme activity, BCA, and ROS levels. (**a**) In vitro free radical scavenging capacity; (**b**) difference in CAT activity of the 5 groups; (**c**) difference in GSH-PX activity of the 5 groups; (**d**) difference in SOD activity of the 5 groups; (**e**) difference in BCA levels of the 5 groups; and (**f**) difference in intracellular ROS levels of the 5 groups. Different letters indicate a significant difference between groups, *p* < 0.05.

In this study, the RRE was evaluated for its in vitro antioxidant activity. The results showed that the RRE exhibited effective in vitro antioxidant properties, as evidenced by its DPPH scavenging activity (EC50 of 0.85 ± 0.01 μg/mL), OH- scavenging activity (EC50 of 1.40 ± 0.04 μg/mL), and, particularly, its ABTS scavenging activity (EC50 of 0.25 ± 0.01 μg/mL), which was the most significant (Figure 3a).

#### 3.3.2. Cellular Antioxidant Enzyme Activity, BCA, and ROS Levels

This study explored the effect of H_2_O_2_ intervention on CAT, GSH-Px, SOD activities, BCA, and ROS levels in HepG2. As shown in Figure 3, when comparing the model group with the control group, CAT (from 10.56 ± 1.03% to 2.90 ± 0.72 U/mg), GSH-Px (from 38.91 ± 3.36 to 12.91 ± 1.42 U/mg), SOD (from 126.50 ± 3.97 to 66.08 ± 6.79 U/mg) enzyme activity, and BCA levels (1020.64 ± 10.01 to 979.36 ± 24.4 μg/mL) were significantly decreased (*p* < 0.05), while ROS levels (from 2,336,120.45 ± 200,123.83 to 5,093,029.41 ± 142,048.49 U/mg prot) were significantly increased (*p* < 0.05). This suggests that H_2_O_2_ intervention in HepG2 cells causes cellular oxidative stress.

This study investigated the changes in these cells under the intervention of TCR by determining antioxidant enzyme activities, BCA levels, and intracellular ROS levels. Compared with the model group, after intervention with TCR, CAT (from 2.90 ± 0.72 to 7.24 ± 0.26 U/mg), GSH-Px (from 12.91 ± 1.42 to 30.57 ± 1.80 U/mg), SOD (from 66.08 ± 6.79 to 99.81 ± 3.65 U/mg) enzyme activities, and BCA levels (from 979.36 ± 24.4 to 972.48 ± 5.61 μg/mL) increased with concentrations, while intracellular ROS levels (from 5,093,029.41 ± 142,048.49 to 3,717,375.02 ± 150,011.44 DCF fluorescence intensity/mg prot) decreased with concentrations. This result indicates that TCR protects H_2_O_2_-induced cellular oxidative damage in HepG2 in a concentration-dependent manner. An increase in antioxidant enzyme activities was observed in the low-concentration group compared with the model, but no significant difference was observed, while significant increases in antioxidant enzyme activities were detected in the moderate- and high-concentration groups compared with the model group (*p* < 0.05). Furthermore, the low- and moderate-concentration groups exhibited significantly decreased BCA levels when compared with the model group, while no significant change was shown in the high-concentration group. This suggests that low and moderate concentrations of RRE may protect oxidized HepG2 cells by inhibiting protein synthesis, while high concentrations of RRE may not have a significant inhibitory effect on protein synthesis.

### 3.4. Metabolite Analysis

#### 3.4.1. Metabolite Composition Analysis

**Figure 4:**
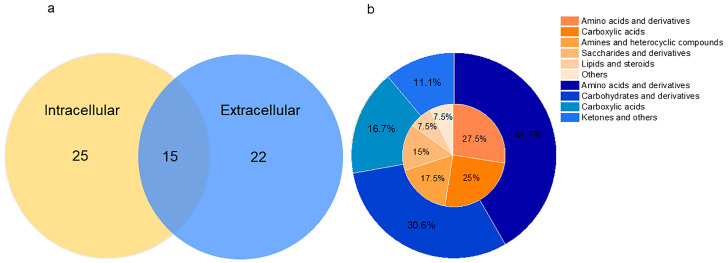
Quantity and composition of intracellular and extracellular metabolites. (**a**) Wayne diagram of the number of extracellular and intracellular metabolites; (**b**) the inner circle represents the proportion of intracellular metabolite composition, and the outer circle represents the proportion of extracellular metabolite composition.

This study used GC-MS to analyze intracellular and extracellular metabolites and identified 62 metabolites. There were 40 intracellular metabolites and 37 extracellular metabolites, of which 15 were common metabolites (Figure 4). The primary composition of intracellular metabolites including amino acids and derivatives (27.50%), followed by carboxylic acids (25.00%), amines and heterocyclic compounds (17.50%), saccharides and derivatives (15.00%), lipids and steroids (7.50%), and others (7.50%). The extracellular metabolites were amino acids and derivatives (40.54%), followed by carbohydrates and derivatives (29.73%), carboxylic acids (16.22%), and ketones and others (10.82%).

#### 3.4.2. Data Pattern and Model Identification

**Figure 5:**
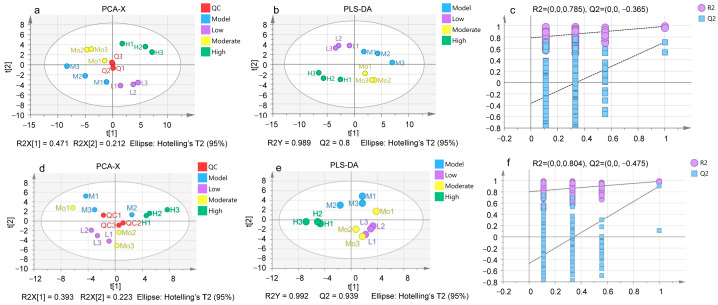
Intracellular and extracellular metabolites PCA, PLS-DA, and permutation test. (**a**,**d**) Visualization of PCA analysis; (**b**,**e**) visualization of PLS-DA analysis; and (**c**,**f**) visualization of the permutation test.

Figure 5a,d shows the plots of PCA analysis for the five groups (intracellular and extracellular). The PCA results indicate that the QC group is positioned centrally on the axes, which suggests the stability of the instrument. The PCA analysis showed that the two principal components explained most of the intracellular and extracellular data, at 68.3% and 61.6%, respectively. There was a separability between the intracellular groups (Figure 5a), indicating that the intervention of TCR altered the intracellular metabolism of H_2_O_2_-induced HepG2 cells. In contrast, extracellularly, the moderate concentration group partially overlapped with both the model group and the low-concentration group, suggesting that the metabolic profiles of the moderate group remained similar to those two groups, while the high-concentration group was clearly separated from the other groups.

PLS-DA (Figure 5b,e) was used to identify the differential metabolites by comparing the experiment groups to the model group. The reliability of the PLS-DA model was confirmed through validation with a 200-permutation test (Figure 5c,f). In the model, R2Y represents how well the model fits the Y matrix, and Cross-Validated Q2 represents the model’s predictive power [39]. The results show that the PLS-DA model possesses an effective fit and predictive ability, as evidenced by R2Y scores of 0.989 and 0.992 and Q2 scores of 0.8 and 0.939, all closely approaching 1. After 200 permutations, the intercept Q2 scores are −0.365 and −0.475, respectively, both of which are less than 0, indicating that the model is not overfitted and confirming the reliability of the analytical results.

#### 3.4.3. Analysis of Differential Metabolites

This study identified 12 intracellular and seven extracellular differential metabolites (*p* < 0.05) by comparing the model group to the TCR groups. The intracellular metabolites are shown in Table 2. In the low-concentration group, octadecanoic acid was up-regulated, while fructose, L-valine, cholesterol, D-mannose, and L-lysine were down-regulated. In the moderate-concentration group, D-glucopyranose, fructose, D-mannose, citric acid, and malic acid were up-regulated, while 1H-purine, cholestenone, and cholesterol were down-regulated. In the high-concentration group, citric acid and propanoic acid were up-regulated, while fructose, L-valine, cholesterol, D-mannose, and L-lysine were down-regulated.

The extracellular metabolites are shown in Table 3. In the low-concentration group, L-alanine, L-proline, L-ornithine, D-glucopyranose, and butanedioic acid were down-regulated. In the moderate-concentration group, L-alanine, L-proline, and D-glucopyranose were down-regulated. In the high-concentration group, L-norvaline, and lactic acid were up-regulated, while l-alanine, L-proline, L-ornithine, and butanedioic acid were down-regulated.

This study also identified the levels of intracellular and extracellular metabolites by comparing the model group to the control group (*p* < 0.05) based on the differential metabolites identified above. The results showed up-regulation of fructose, D-mannose, and L-lysine and down-regulation of citric acid, malic acid, propanoic acid, and cholesterol. Extracellularly, D-glucopyranose was up-regulated, while L-norvaline and lactic acid were down-regulated.

#### 3.4.4. Differential Metabolite-Enriched KEGG Pathways

**Figure 6:**
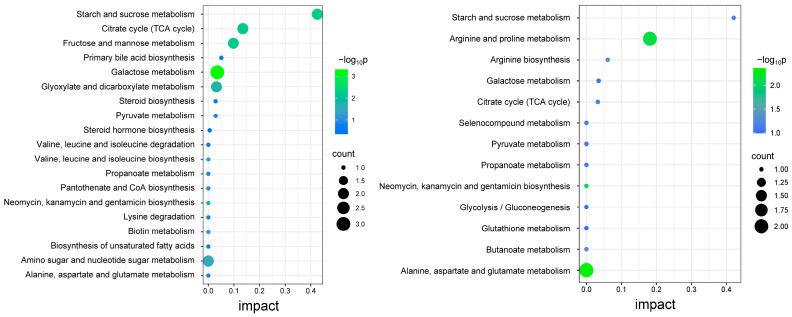
Intracellular (**left**) and extracellular (**right**) differential metabolite-enriched KEGG pathway. The horizontal coordinates represent the impact value for each pathway; the vertical coordinates represent the pathways enriched with differential metabolites. The size of the points indicates the number of metabolites in the enriched pathways, and the colors represent the significance, expressed as −log_10_ (p).

Figure 6 shows the results of the KEGG pathway analysis of intracellular and extracellular differential metabolites, where the horizontal coordinates represent the impact value. The impact value was obtained by normalization, using the degree centrality and the betweenness centrality metrics for each metabolite node [37]. Based on a statistical significance of *p* < 0.05, five major KEGG metabolic pathways were identified in both the intracellular and extracellular differential metabolites. These differential metabolites were intracellularly enriched in starch and sucrose metabolism, the tricarboxylic acid cycle (TCA cycle), fructose and mannose metabolism, primary bile acid biosynthesis, and galactose metabolism. Extracellularly, they were enriched in starch and sucrose metabolism, arginine and proline metabolism, arginine biosynthesis, galactose metabolism, and the tricarboxylic acid cycle (TCA cycle).

## 4. Discussion

Growing evidence indicates the pivotal role of ROS in promoting the development and progression of various diseases, suggesting the need to find antioxidant strategies that fulfill both safety and effectiveness. In this background, the natural RR stood out because of its combination of safety, nutritional value, and significant antioxidant capacity. In this study, we quantified the nutritional and bioactive components in RRE and elucidated the antioxidant properties of RRE and its protective effects on a HepG2-based oxidative damage cell model through in vitro and cellular experiments. Furthermore, the results suggested that its antioxidative effects may be related to increased antioxidant enzyme activities and BCA levels and decreased ROS levels. Changes in intracellular and extracellular metabolite levels were identified by metabolomics analysis in H_2_O_2_-induced HepG2 cells following RRE intervention.

### 4.1. Bioactive Components

This study quantified phenolic compounds, proteins, amino acids, organic acids, polysaccharides, and SOD. The results showed a high concentration of phenolics and flavonoids in the RRE. Consistent with this finding, previous studies have shown that more than 20 flavonoids have been isolated from RR fruits, including quercetin, kaempferol, myricetin, and catechin, among others [32]. Moreover, the total flavonoid content in RRE was higher than that in blueberries (1.6 ± 0.3 mg RE/g) [40]. Plant polyphenols and flavonoids are known for effective antioxidant and anticancer activities, and their antioxidant mechanisms include electron supply, metal ion chelation, ROS scavenging, and the inhibition of ROS-promoting enzymes [8,9,10,11,12,13,14,15,16,17,18,19,20,21,22,23,24,25,26,27,28,29,30,31,32,33,34,35,36,37,38,39,40,41]. Consistently, in a previous study, Chen et al. [42] found that quercetin in RR significantly increased the levels of antioxidant factors Nrf2, HO-1, and NQO1 in the brain tissues of D-galactose-induced aging mice and enhanced antioxidant capacity by activating the antioxidant-related Nrf2 pathway.

In contrast to the results of Min et al. for the amino acid composition of three RR genotypes, tryptophan was not detected in our RRE [43]. In terms of organic acids, this study did not detect malic acid, citric acid, or tartaric acid, which have been reported in RR by Su et al. [44]. Differences in amino acid and organic acid may be caused by different extraction processes or differences in plant genotypes. In particular, a study by Huang et al. [33] indicated that RR contains highly abundant L-ascorbic acid (~1300 mg/100 g), whereas we reported that the content of L-ascorbic acid in RRE was ~662.46 mg/100 g. This difference may be attributed to the decrease in the content of chemically active ascorbic acid due to its oxidation during drying of RR [33]. However, with the loss of L-ascorbic acid, the amount detected in RRE was still about 6 times higher than that of kiwifruit (*Actinidia deliciosa*, 105 mg/100 g) [45], which is commonly recognized for its high ascorbic acid content. L-ascorbic acid has been shown to have high antioxidant properties [14,46]. It enhances the body’s antioxidant capacity by neutralizing free radicals, reacting with oxidized forms of vitamin E, and promoting the activity of antioxidant enzymes [14]. This potent antioxidant effect is demonstrated by its ability to protect important biomolecules from damage by oxidants and external toxins and pollutants [14].

Previous studies have demonstrated varying functionalities of RR polysaccharides, including antioxidant and anti-tumor effects. Wang et al. found that RR polysaccharides (RTFP-50 and RTFP-80) exhibited strong in vitro antioxidant capacity and inhibited α-glucosidase [47]. In addition, Wang et al. showed that RR polysaccharides could enhance the antioxidant capacity of db/db mice by increasing antioxidant enzyme activities and lowering MDA levels [48]. However, contrasting results were indicated by Jin et al. [49], who showed that a different RR polysaccharide (RTFP-1) promotes apoptosis and increases ROS levels in HepG2 cells, suggesting potential anti-tumor applications.

Furthermore, the results showed that the dried RRE still retained a high SOD activity. It has been reported in a previous study that RR is one of the fruits known to have the highest SOD activity, with an SOD activity 20–50 times higher than that of grapes [24]. SOD is an enzyme that converts superoxide anion radicals (O2−) into less toxic oxygen (O_2_) and H_2_O_2_, thereby reducing oxidative damage to cells and proteins by free radicals and exerting an antioxidant effect [50]. Therefore, RR is recognized as a potential natural antioxidant because of its rich content of antioxidant-active components.

### 4.2. Antioxidative Activity and Protective Effect of RRE on HepG2 Cells

Based on the abundant antioxidant active components in the RRE, further experiments were conducted to assess its antioxidant capacity in vitro. The results showed that the RRE exhibited significant in vitro antioxidant activity in scavenging DPPH radicals, ABTS radical cations, and hydroxyl radicals. This finding is supported by Yan et al. [51], who indicated the antioxidant effect of RRE was primarily in the scavenging capacity of DPPH radicals and ferric iron reduction capacity. In addition, dried RR had better antioxidant activity in DPPH and FRAP scavenging capacity compared with fresh RR [51]. Furthermore, Jiang et al. indicated that total flavonoids and the total phenolic acid content were significantly and positively correlated with ABTS radical cation scavenging capacity (*p* < 0.05) [52]. However, the reported amino acid content showed a significant negative correlation with ABTS scavenging capacity (*p* < 0.05), a result that warrants further investigation [52].

To further explore the cellular antioxidant effects of RRE, HepG2 cells were used in this study. Primary hepatocytes (PHHs) are highly differentiated cells that encounter challenges such as a rapid decline in cell viability and phenotypic instability in an in vitro environment, whereas HepG2 cells are more easily cultured and passaged than PHHs and retain some metabolic characteristics of liver cells, making it a commonly used cell model for liver metabolomics studies [53]. The results showed that TCR had a significant protective effect against H_2_O_2_-induced HepG2 cell damage. The mechanism of this protective effect may be attributed to the fact that RRE significantly increased the activities of CAT, GSH-Px, and SOD and levels of BCA and decreased the levels of ROS. This effect showed a concentration-dependent pattern. Several previous studies also support our findings. Xu et al. [54] reported that RR extracts increased the antioxidant enzyme activities of GSH-Px, T-SOD, and CuZn-SOD in mouse liver homogenates and improved cell viability. Ni et al. [55] showed that five different varieties of RR extracts had significant protective effects against H_2_O_2_-induced HaCaT cell damage, as evidenced by SOD and GSH-Px activities, as well as a decrease in malondialdehyde (MDA) content. Li et al. [56] found that RRE was able to significantly down-regulate ROS levels in normal human colonic epithelial cells (NCM460) induced by dextran sulfate sodium (DSS) and up-regulate SOD1, SOD2, and GSH-Px2 mRNA expression. Briefly, these results suggest that RRE, as a potential natural antioxidant, exhibits a broad antioxidant property, both in vitro and at the cellular level.

### 4.3. Differential Metabolite Analysis

This study delved into the effects of RRE on metabolic regulation in the HepG2 cell model. Our results showed that the RRE was able to regulate important metabolites in several metabolic pathways, including glucose-related metabolism, citric acid metabolism, and primary bile acid biosynthesis, in HepG2 cells exposed to oxidative stress.

Our results showed that the RRE up-regulated metabolites related to glucose-related metabolism. HepG2 cells exhibit a distinctive pattern of metabolic adaptation in oxidative stress environments, known as the Warburg effect, which preferentially uses aerobic glycolysis over oxidative phosphorylation as the primary energy production pathway [57]. In order to maintain intracellular oxidative stress homeostasis, cells rely on a series of antioxidant enzymes, including CAT, GSH-Px, and SOD, which are composed of specific protein structures to catalyze the catabolism of oxidative products and neutralize ROS, and whose generation and functioning are both dependent on an adequate energy supply. Previous studies showed that treatment of HepG2 cells with 500 μmol/L H_2_O_2_ significantly increased cellular glucose uptake, suggesting that under oxidative stress conditions, cells mitigate oxidative stress by regulating glucose metabolism in response to energy demand [58]. In contrast, the up-regulation of glucose metabolism-related metabolites in oxidatively stressed HepG2 cells after the intervention of RRE, as well as the observed increase in the activities of three types of enzymes in this study, can be attributed to the positive regulation of energy metabolism by RRE. In turn, it supported the increase in the activities of antioxidant enzymes. Although high glucose metabolism levels are usually accompanied by oxidative stress as well, we hypothesized that the efficiency of ROS scavenging under the protection of a concentration of RRE was higher than the efficiency of generation in a high-glucose cellular environment, as evidenced by the decrease in ROS levels after RRE intervention.

At specific concentrations, RRE may promote cellular antioxidants by upregulating citric acid and malic acid metabolites in the TCA cycle. This may be because these acids contributed to the restoration of the TCA cycle and metabolic reprogramming, which improved mitochondrial function in H_2_O_2_-damaged HepG2 cells. Cancer cells are usually accompanied by dysfunctional mitochondria, leading to decreased efficiency of the TCA cycle, which promotes ROS generation in the electron transport chain [59]. This phenomenon arises from the leakage of electrons in Complex I and Complex III, which promotes the conversion of oxygen into superoxide (O2−), exacerbating oxidative damage to cells [59]. Moreover, H_2_O_2_ intervention further exacerbates the hypoxic dilemma of cancer mitochondrial function with increased ROS levels. Previous studies have found that the TCA cycle is still present in cancer cells, but it is usually reprogrammed to the Citrate Shuttle and Malate–Aspartate Shuttle to support the metabolic needs of cancer cells [60]. Citric acid is transported to the cytoplasm via the Citrate Shuttle, producing the downstream product malate for return to the mitochondria [61]. Malic acid can also be returned to the mitochondria via the Malate–Aspartate Shuttle, helping to maintain the NADH/NAD+ ratio in the cell [61]. Maintaining these shuttle mechanisms not only supports metabolic demands in the cytoplasm but also restores H_2_O_2_-infected mitochondrial function by regulating metabolite levels and the redox state within the mitochondria [61]. More studies are needed to explore the antioxidant function of citric acid at the molecular level in the future.

Citric acid plays a significant role in regulating and maintaining redox homeostasis within liver tissues, reducing lipid peroxidation product accumulation while exhibiting hepatoprotective, apoptosis-inhibiting, and anti-inflammatory potential [62]. In Kim et al.’s [63] study of hepatic ischemia–reperfusion injury in a rat model, it was found that administering a moderate amount of citric acid (100 mg/kg) significantly enhanced antioxidant enzyme activity and effectively inhibited oxidative stress. Similarly, orally administered citric acid increased hepatic GSH-Px activity and inhibited the toxic effects of nitric oxide production and its reaction with superoxide anion to generate peroxynitrite (ONOO-) in a bacterial lipopolysaccharide-induced mice model, further protecting the cells from oxidative damage [64]. Furthermore, a study by Zhou et al. [65] reported that citric acid reduced ROS levels in normal human epidermal melanocytes (NHEMs) in a concentration-dependent manner ranging from 0 to 10 mM, but it conversely increased ROS levels in mouse cutaneous melanoma cells (B16F10). However, the effect of intracellular citrate is dose-dependent. Petillo et al. [66] reported that a ≤5 mM concentration of citrate had no significant effect on HepG2 cell viability. In contrast, Chen et al. [67] found that high doses (120 mg/kg peritoneally per week for 3 weeks) of citric acid decreased T-SOD and GSH-Px activity, causing oxidative damage to the liver, as evidenced by increased MDA levels. These results suggested that appropriate concentrations of citric acid might regulate oxidative stress by decreasing the levels of lipid peroxidation and enhancing the activity of antioxidant enzymes, thereby reducing the generation of ROS. Additionally, citric acid may balance intracellular oxidative stress homeostasis by restoring mitochondrial function. However, the antioxidant effect of citric acid is dose and receptor-dependent.

RRE may restore cell membrane fluidity by reducing cholesterol levels, thereby maintaining oxidative stress homeostasis. Cholesterol is a key component of the structures that make up the lipid bilayer of cell membranes, and its dynamic homeostasis is essential for maintaining cellular function integrity and membrane fluidity [68]. The regulatory mechanisms of cholesterol include processes such as de novo synthesis, uptake of exogenous cholesterol via membrane transport proteins, sterol ester storage, and cholesterol efflux [68]. However, under pathological conditions, such as the development of hepatocellular carcinoma, cholesterol tends to accumulate abnormally in cancer cells [69]. Excessive cholesterol accumulation is a factor in the generation of ROS, which disrupts the fluidity of the mitochondrial membrane, leading to the dysfunction of membrane proteins and affecting the transmembrane transport of key carrier proteins [69]. Our results indicated that cholesterol concentrations decreased significantly after treatment with H_2_O_2_ (2 mmol/L), and the intervention of TCR further contributed to this decrease. However, the results of Seo et al. [58] reported that intervention in HepG2 cells using low concentrations (0.5 mmol/L) of H_2_O_2_ up-regulated the expression of caveolin-1 and LDLR, thereby promoting cholesterol synthesis and lipid accumulation. This suggests that different concentrations of H_2_O_2_ treatment have different effects on cholesterol metabolism in HepG2 cells. The high concentration (2 mmol/L) of H_2_O_2_ and all TCRs down-regulated intracellular cholesterol levels, whereas the low concentration of H_2_O_2_ may further promote cholesterol accumulation. The results of Zhang et al.’s [70] study were similar to our results, which showed that RR juice decreased oxidized LDL formation in mouse macrophage foam cells while significantly inhibiting intracellular cholesteryl ester accumulation by promoting intracellular cholesterol efflux. In addition, Tan et al. [29] showed that RR ellagitannin significantly reduced LDL-C concentration in the db/db mouse liver, further suggesting that RRE possesses the ability to regulate cholesterol synthesis. Based on the above background, RRE may regulate intracellular ROS levels by down-regulating cholesterol concentration, thereby exerting its potential antioxidant effects.

Finally, this study identified the role of RRE in regulating primary bile acid biosynthesis in oxidatively stressed HepG2 cells. In vitro studies have shown that the excessive accumulation of bile acids (BAs) induces mitochondrial dysfunction and promotes the generation of ROS, thereby triggering a cytotoxic response [71]. For example, bile acids like taurodeoxycholic acid, glycocholic acid, glycolichocholic acid, and deoxycholic acid have demonstrated the capability to trigger the generation of ROS and induce cell apoptosis in isolated rat hepatocytes as well as human hepatocellular carcinoma cells [71,72]. RRE regulates the classic bile acid synthesis pathway by down-regulating the intracellular levels of cholesterol and its metabolic intermediate cholestenone, which was reported in a study by Ma et al. [73] based on a Wistar rat model. Ma et al. [73] reported the potential protective role of RRE juice in alleviating arsenic-induced hepatic injury. Specifically, RRE not only significantly restored the level of S-adenosylmethionine (SAM) in arsenic-induced Wistar rats but also indirectly inhibited hepatic bile acid synthesis by restoring the negative feedback regulation mechanism dependent on the histone H3K36 trimethylation (H3K36me3) [73].

## 5. Conclusions

Our results indicated that RRE exhibits effective antioxidant properties both in vitro and in H_2_O_2_-induced HepG2 cells. This property is attributed to the existence of diverse antioxidant bioactive substances and boosting cellular SOD, CAT, and GSH-Px activities and BCA levels while reducing ROS levels. Metabolomics analysis further indicated the restoring cell viability effects of RRE at different concentrations, revealing RRE-regulated cellular metabolites, thereby affecting both intracellular and extracellular metabolic pathways. RR’s abundance of natural antioxidant components and its favorable antioxidant properties make it promising for functional food and nutraceutical applications. Foods and dietary supplements with RRE as an additive can provide consumers with a natural and effective way to enhance the intake of antioxidants. Additionally, although this study used the HepG2 cell line to characterize complex metabolic changes, there is a necessity to use alternative cell lines to elucidate the complex metabolic networks following RRE intervention in the future.

## Figures and Tables

**Table 1:** Active substance concentrations.

Concentration	Mean ± SD
Total phenol (μg GAE/mL)	793.83 ± 12.74
Total flavonoid (μg RE/mL)	5098.88 ± 39.03
Soluble protein (μg/mL)	115.80 ± 5.25
Total amino acids (μg/mL)	428.33 ± 3.26
Total organic acids (mg/mL)	43.82 ± 0.23
Total sugar (mg/mL)	15.29 ± 0.25
Polysaccharide (mg/mL) SOD (U/mL)	4.75 ± 0.86 81.74 ± 0.54

**Table 2:** Intracellular differential metabolites.

Metabolites	VIP	Model/Control	Low/Model	Moderate/Model	High/Model
D-Glucopyranose	1.42182	N/A	N/A	↑	N/A
Fructose	1.36174	↑	↓	↑	↓
Citric acid	1.20151	↓	N/A	↑	↑
Malic acid	1.18358	↓	N/A	↑	N/A
Octadecanoic acid	1.18167	N/A	↑	N/A	N/A
L-Valine	1.17616	N/A	↓	N/A	↓
Propanoic acid	1.16973	↓	N/A	N/A	↑
1H-Purine	1.14514	N/A	N/A	↓	N/A
Cholestenone	1.09774	N/A	N/A	↓	N/A
Cholesterol	1.08222	↓	↓	↓	↓
D-Mannose	1.03012	↑	↓	↑	↓
L-Lysine	1.00687	↑	↓	N/A	↓

N/A means no significant differences (*p* > 0.05); ↑ means up-regulated; ↓ means down-regulated.

**Table 3:** Extracellular differential metabolites.

Metabolites	VIP	Model/Control	Low/Model	Moderate/Model	High/Model
L-Alanine	1.37156	N/A	↓	↓	↓
L-Proline	1.2146	N/A	↓	↓	↓
L-Ornithine	1.20095	N/A	↓	N/A	↓
D-Glucopyranose	1.12173	↑	↓	↓	N/A
L-Norvaline	1.1202	↓	N/A	N/A	↑
Lactic acid	1.05977	↓	N/A	N/A	↑
Butanedioic acid	1.00187	N/A	↓	N/A	↓

N/A means no significant differences (*p* > 0.05); ↑ means up-regulated; ↓ means down-regulated.

## Data Availability

The original contributions presented in the study are included in the article, further inquiries can be directed to the corresponding author.
